# 

**Published:** 1896-09

**Authors:** 


					﻿NOTICE.
JtU «r*4eZe« for publication, books for review, business communications, etc.,
mult be sent to Editor, 1231 Locust Street, Philadelphia.
Subscribers will please notice that this Journal will be sent until arrears
are paid and it is ordered discontinued.
				

